# Recovery of Transient High-Grade Atrioventricular Block Managed With Corticosteroid Therapy Without Device Implantation in Newly Diagnosed Cardiac Sarcoidosis: A Case Report

**DOI:** 10.7759/cureus.41481

**Published:** 2023-07-06

**Authors:** Azeem Rathore, Okechukwu Mgbemena, Pedro Adrover Lopez, Prakash Suryanarayana, John N Catanzaro, Stephen Keim

**Affiliations:** 1 Internal Medicine, University of Florida College of Medicine - Jacksonville, Jacksonville, USA; 2 Cardiology, University of Florida College of Medicine - Jacksonville, Jacksonville, USA; 3 Electrophysiology, University of Florida College of Medicine - Jacksonville, Jacksonville, USA

**Keywords:** case report, implantable cardioverter-defibrillator, pacemaker, device implantation, corticosteroid therapy, high-grade atrioventricular block, cardiac sarcoidosis

## Abstract

Atrioventricular blocks (AVBs) presenting in cardiac sarcoidosis (CS) remain an ongoing challenge for clinicians. While most initiate immunosuppressive therapy with the goal of pursuing device implantation, there is some ambiguity as to which patient cohorts actually benefit from device therapy. We present a case of a 39-year-old African American male with a past medical history of hypertension and no prior cardiac history who presented with substernal chest pain in the setting of a hypertensive emergency. He was later diagnosed with cardiac sarcoidosis by cardiac magnetic resonance imaging. His hospital course was complicated by transient Mobitz II atrioventricular block. He was started on prednisone, and while initially scheduled for an implantable cardioverter-defibrillator (ICD), his conduction block recovered. Through a multidisciplinary approach, the patient was discharged on medical management with outpatient follow-up. Since his initial hospitalization, the patient has not had any concerning cardiovascular events over the past year and has not been treated with device therapy. Our case illustrates the feasibility of effectively managing patients with cardiac sarcoidosis presenting with transient atrioventricular blocks only with corticosteroid therapy without needing device implantation.

## Introduction

Sarcoidosis is a chronic condition with an unknown cause that is marked by non-necrotizing granulomatous inflammation affecting multiple organs and systems, including the heart [1]. Estimated to account for 5% of patients with systemic sarcoidosis, cardiac sarcoidosis (CS) is a rare but serious condition in which inflammation and granulomas develop in the heart tissue [2]. Initially thought to be fairly uncommon, the prevalence of CS has likely been underestimated with recent data indicating that over 25% of sarcoid patients may demonstrate evidence of cardiac involvement based on autopsy or imaging studies [2]. The presence of granulomas can interfere with the normal electrical signals that control heartbeats, leading to the sequela of conduction abnormalities, ventricular arrhythmias, supraventricular tachyarrhythmias, cardiomyopathy, and sudden cardiac death (SCD) [1,2]. As it relates to conduction abnormalities, atrioventricular block (AVB) requiring a permanent pacemaker (PPM) or implantable cardioverter-defibrillator (ICD) is the most common initial manifestation of CS [3].

In terms of management strategy, corticosteroids are considered the primary mainstay therapy for clinically active or symptomatic CS and have shown a reasonable chance of AV nodal conduction recovery [4-6]. However, there is a lack of established guidelines or reference standards to determine the optimal timing, duration, or intensity of immunosuppressive therapies for this cohort of patients [7]. Despite the limited evidence, corticosteroids continue to be considered the primary treatment for clinically active or symptomatic CS and are recommended by the majority of experts. However, atrioventricular (AV) nodal recovery is unpredictable and can be transient, which likely explains why the Heart Rhythm Society (HRS) guidelines highly encourage device therapy due to the potential risk of malignant arrhythmias in these patients [8]. In the absence of randomized controlled trial data, HRS guidelines are mainly supported by retrospective and prospective small cohort studies as well as case report literature for their recommendations. However, since the inception of the 2014 HRS guidelines, there has been growing discussion that there may be potential overtreatment with device therapy in patients with CS complicated by symptomatic AVB [6]. Specifically, some authors have argued that early initiation of corticosteroid therapy without device implantation may be a potential therapeutic approach for carefully selected patients with symptomatic AVB [9,10]. We present a case of newly diagnosed CS with concomitant transient type II Mobitz AVB in a patient that was successfully managed with corticosteroid therapy without ICD implantation and without any concerning cardiovascular events in the ensuing year.

## Case presentation

A 39-year-old African American male with a past medical history of uncontrolled hypertension presented to the emergency department for sudden onset of chest pain one hour before arrival. While sitting in his bed, the patient described experiencing a severe, substernal “chest tightness” without radiation that lasted one hour in duration. He additionally endorsed diaphoresis and left-handed numbness. During that time, he took two low-dose aspirins without any relief of symptoms. He reported no prior history of similar symptoms and no family history of cardiac disease and denied any alcohol, tobacco, or drug abuse. On presentation, his vitals were noted as blood pressure of 230/112 mmHg, pulse rate of 71 bpm, 100% oxygen saturation on ambient air, respiratory rate of 17, and a 98.8°F temperature. His physical examination was unremarkable.

**Figure 1:**
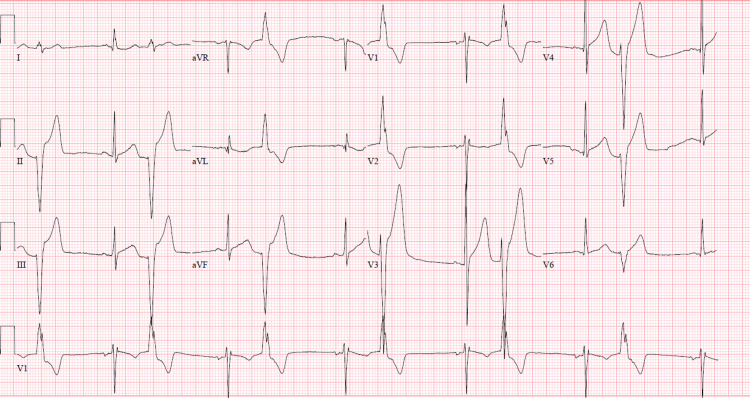
ECG depicting bigeminy with RBB morphology and a superior axis; PVCs likely are originating from the septal wall or apex of the left ventricle. ECG: electrocardiogram, RBB: right bundle branch, PVC: premature ventricular complex

**Figure 2:**
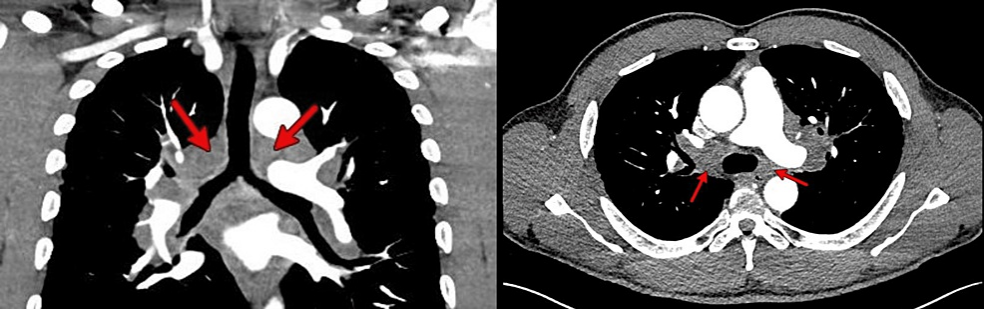
Chest CTA showing bilateral hilar and mediastinal lymphadenopathy with bilateral pulmonary perilymphatic nodules in cross-sectional (left) and transverse (right) views. CTA: computed tomography angiography

An initial electrocardiogram (ECG) showed premature ventricular complexes (PVCs) with a bigeminy pattern along with a right bundle branch (RBB) morphology with a superior axis (Figure 1). Laboratory data included a complete blood count and comprehensive metabolic panel that was within normal limits. High-sensitivity cardiac troponin levels (normal values: <14 ng/L) were serially measured with values of 32 ng/L, 490 ng/L, and 1,080 ng/L at times zero, one hour, and three hours, respectively, and with a delta of 590 ng/L between zero and three hours. Of note, neither C-reactive protein nor erythrocyte sedimentation rate was obtained. A chest radiograph showed signs of hilar lymphadenopathy, and a follow-up computed tomography angiography (CTA) of the chest revealed bilateral hilar and mediastinal lymphadenopathy (Figure 2). The patient was admitted to the cardiovascular care unit and started on heparin and nitroglycerin infusions for the management of hypertensive emergency in the context of suspected non-ST segment elevation myocardial infarction or type II myocardial ischemia.

**Figure 3:**
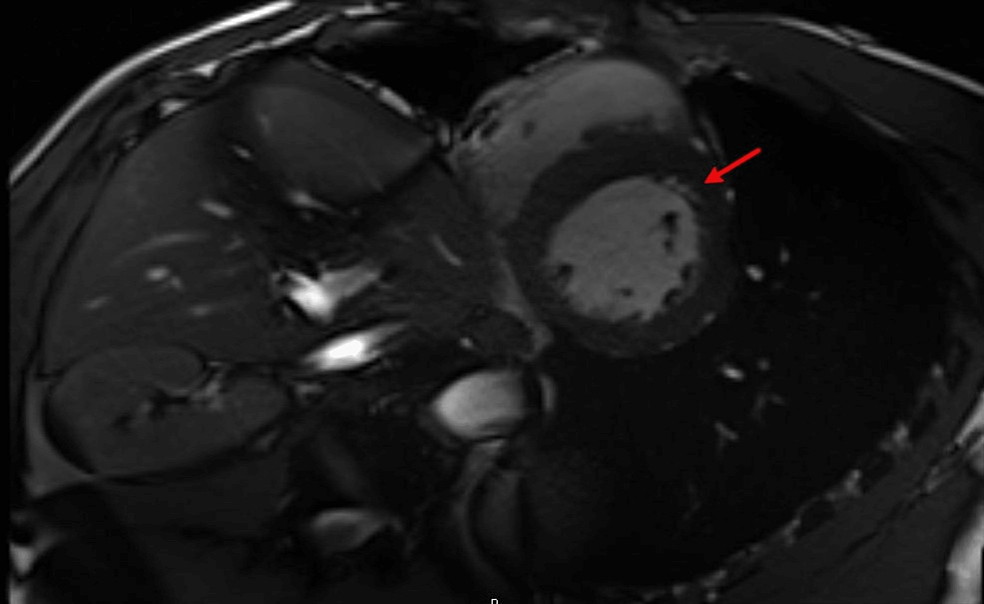
Focal LGE in the mid-myocardium and right ventricular side subendocardium of the interventricular septum at the level of mid-ventricle and apex. LGE: late gadolinium enhancement

**Figure 4:**
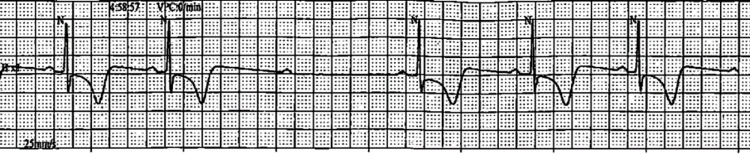
Cardiac telemetry on day 3 showing a transient Mobitz II AVB. AVB: atrioventricular block

Initial diagnostic workup included a transthoracic echocardiogram (TTE) that revealed a left ventricular (LV) ejection fraction of 55% along with mid-distal septal hypokinesis consistent with the ventricular ectopy localized in his ECG (Video 1). Due to the CTA chest results, a cardiac magnetic resonance imaging (CMR) test was performed, which revealed evidence of late gadolinium enhancement (LGE) consistent with myocardial fibrosis or scarring suggestive of CS (Figure 3). A few transient Mobitz II AVBs were noted on telemetry, although only one strip was documented and retained for review (Figure 4).

**Video 1:** Transthoracic apical four-chamber view showing mid-distal septal hypokinesis consistent with suspected localization for PVC ectopy; left ventricular ejection fraction of 55%. PVC: premature ventricular complex

Following a collaborative approach involving electrophysiology (EP) and pulmonology, the decision was made to initiate a daily prednisone regimen of 40 mg due to the uncertain prognosis associated with high-degree AVB. Additionally, an implantable cardioverter-defibrillator (ICD) was tentatively scheduled to be implanted during this admission as well. In the interim, considering the suspicion of extracardiac sarcoidosis, bronchoscopy with biopsy confirmed the presence of non-caseating granulomas consistent with sarcoidosis. Of note, the diagnosis of CS was made based on the HRS guidelines previously mentioned [8].

**Figure 5:**
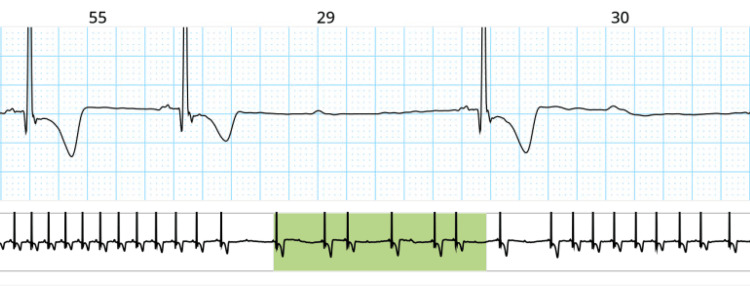
Transient Mobitz II AVB identified on Holter monitoring; heart rate of 35 bpm. AVB: atrioventricular block, bpm: beats per minute

Over the next 48 hours, no additional conduction abnormalities were observed on telemetry. After a shared decision-making process, the patient opted for medical management instead of device implantation and was consequently discharged with a tapered dose of oral steroids and strict outpatient follow-up. However, approximately two months later, the patient returned due to another hypertensive emergency episode resulting from medication noncompliance in the context of several missed appointments. A coronary angiogram performed during that admission indicated patent coronary arteries. Following the second hospitalization, the patient started regular follow-ups and monitoring with his providers. One month after his second hospitalization, Holter monitoring revealed a recurrence of the transient Mobitz type II AVB (Figure 5). The patient was continued on a low-dose prednisone regimen for one year before tapered discontinuation. At the one-year mark, a repeat TTE revealed no worsening of his ejection fraction. As of this writing, no further remote patient monitoring has been administered, a positron emission tomography (PET) scan has been deferred, and no EP study has been performed as of yet on the patient.

## Discussion

The clinical manifestations of CS include advanced-degree heart block, atrial tachycardia, ventricular arrhythmias, and heart failure [4]. Of the various conduction abnormalities, high-grade AVBs are the most common and have been previously associated with poor outcomes [3,4]. For example, two prominent studies previously showed that the risk of fatal and non-fatal adverse cardiac events, such as SCD, was significantly elevated in CS patients presenting with high-grade AVB, which likely explains why most clinicians prefer urgent device implantation [3,11]. The HRS guidelines on arrhythmia management in CS specifically provide a class IIa recommendation that encourages device implantation in patients with AVBs [8]. Therefore, despite the transient nature of the recognized Mobitz II AVB, we strongly believed that urgent ICD implantation was the appropriate subsequent management step especially due to the well-established mortality risks associated with high-grade AVBs in CS patients.

Although various society guidelines recommend device implantation for patients with CS complicated by AVB, there exists an ongoing debate regarding the necessity of device therapy in this specific patient population [10,12]. A recently published retrospective study, for instance, evaluated the efficacy of steroid therapy alone in the treatment of AVB in CS without seeking PPM or ICD implantation [9]. Out of a study population of 10 patients, three patients presented with transient AVB, of which two recovered to a 1:1 AV conduction after initiation of steroid therapy without pursuing device therapy. Specifically, a 79-year-old female had recovery of a transient Wenckebach block and had an uneventful clinical course over the next five years; the other case involved a 59-year-old male with recovery of a transient 2:1 AVB that also had no clinical events over the ensuing two years. The remaining third patient showed no improvement in AV conduction and required PPM implantation. Furthermore, the other seven patients with CS had concomitant third-degree AVB, and all required device therapy before starting steroid therapy mainly because of hemodynamic instability. In comparison, our patient in the ensuing year has had no concerning cardiovascular events after exclusively being managed with corticosteroid therapy. In another case, authors described a 65-year-old female with CS who presented with 2:1 AVB that recovered to normal sinus rhythm after starting prednisolone, and subsequently, PPM implantation was not pursued in this case as well [13]. With regard to transient AVB, authors of a 2018 case described a patient with newly diagnosed CS complicated by intermittent complete AVB that recovered after two weeks of prednisolone 30 mg daily who also did not receive device implantation [14]. Finally, there have been a few cases reported whereby clinicians have administered high-dose steroid therapy, also known as pulse steroid therapy, for cases of complete AVB that lead to recovery as well and again without pursuing device therapy [15,16]. Thus, as previous cases and clinicians have discussed, there may be a cohort of CS patients with high-grade AVB that can optimally be managed with steroid therapy alone without needing urgent device implantation [10,12].

Indeed, there likely exists potential overtreatment with device therapy in some patients with active CS presenting with conduction abnormalities. Early initiation of immunotherapy, potentially at higher initial doses and perhaps even in combination with other anti-inflammatory agents, might significantly reduce the need for PPM or ICDs. Obviously, the risk of a transient AVB recurring and possibly degenerating into a ventricular arrhythmia lies at the center of what clinicians must weigh versus a more conservative approach of only immunosuppressive therapy. Moreover, there would also be a commensurate decrease in device-related complications given the presence of scarring, fibrosis, active inflammation, and immunosuppression with steroids in CS patients [11]. Importantly, clinical follow-up appears mandatory if immunotherapy alone is the management strategy for this cohort of patients, which was the case with our patient given close monitoring over the ensuing year.

As previously noted, while some authors have argued that the possibility of disease progression should warrant ICD implantation even if the high-grade or complete AV conduction block transiently recovers, this stance appears to be challenged by emerging case report literature [12-16]. Our patient’s successful response to steroid therapy, though, should not be entirely surprising given that his patient profile was consistent with known factors associated with potential improvement in response to therapy in CS patients presenting with AVBs [11,12]. Specifically, early treatment, younger age, and preserved LV systolic function are reported to have higher rates of AVB recovery for which our patient met all characteristics. However, our patient had some features that have been associated with increased mortality and worse outcomes: the presence of a high-grade AVB, LGE noted on CMR, and elevated troponins on admission [10]. Notably, the presence of LGE on CMR in the setting of normal LV function for our patient would be a class IIa recommendation according to HRS guidelines for an electrophysiology study (EPS) to assess candidacy for device therapy as recovery of AVB may not be expected [8]. As mentioned before, the patient has not received an EPS and remains asymptomatic as well. Further, most cases of AVB recovery on steroid therapy have occurred in patients with normal LV dysfunction like our patient [9,14,15]. Thus, while the initial management strategy included both steroid therapy and ICD implantation, recovery of his transient AVB, as well as the patient’s reluctance toward an invasive procedure, led to a shared decision to continue with only steroid therapy so long as the patient had a reliable follow-up with the proper specialists. While the patient was briefly lost to follow-up in the two months following his discharge, he reliably followed up with his various providers over the ensuing year without having any concerning cardiovascular events.

In the absence of large, randomized trials or prospective registries of patients with CS, organizations such as HRS, the European Respiratory Society (ERS), and the Japanese Ministry of Health and Welfare (JMHW) will need to continue relying on retrospective studies and case reports to inform recommendations [8,17,18]. While there have been a few prominent systematic reviews evaluating the efficacy of corticosteroid therapy on CS within the past two decades, there has yet to be a comprehensive review of whether certain patients can benefit from steroid therapy alone without device therapy and also be prospectively followed to monitor for any conduction abnormalities or malignant arrhythmia events [4,5]. Further data is required to help clinicians identify if certain patients with CS can optimally be treated with steroid therapy without the need for PPM or ICD.

There are some limitations with this case worth noting. Firstly, while the patient in the year since his initial CS diagnosis had no reoccurrence of symptoms, he never was evaluated with an EPS nor a PET scan, which are strongly recommended by the HRS [8]. Secondly, instead of a high-grade AVB, it could be argued that the patient suffered from transient vagal-mediated AVB, which would be explained by a different mechanism: either from inflammation and granulomas of the vagus nerve or, more likely, compression from the mass effect of the large mediastinal adenopathy. However, vagal-mediated AVB is characterized by sinus node slowing with concomitant gradual lengthening of the PR interval, which was absent in the lone recorded telemetry strip in our patient. Moreover, some have argued that vagal-mediated AVBs may progress to more advanced forms of heart block and thus may be considered as a potential early manifestation of advanced conduction disease, although the indication for device therapy is generally not indicated [19]. Finally, while additional dropped beats were observed during the monitoring period, only one telemetry strip was recorded and printed for review, and thus, additional evidence for an intra-Hisian disease is lacking, which could have otherwise been elucidated via EPS. Still, the initial recommendation was ICD implantation given the patient’s risk factors and overall clinical presentation.

## Conclusions

This case report highlights a middle-aged male with newly diagnosed CS complicated by a transient high-grade AVB that was effectively managed with steroid therapy without requiring ICD implantation. The patient had no known history of cardiac disease, and his presentation was initially consistent with a non-ST segment elevation myocardial infarction or type II myocardial ischemia. However, the diagnosis of CS was suspected based on the chest CT, which showed bilateral hilar and mediastinal lymphadenopathy, and CMR, which showed LGE. The patient’s hospitalization was complicated by a transient Mobitz II AVB that was managed with prednisone therapy without device therapy. There is an emerging literature that challenges the conventional approach of pursuing device implantation in CS patients with symptomatic high-grade AVB when corticosteroid therapy may feasibly be efficacious in terminating the conduction abnormalities. However, additional data is needed to determine the efficacy of corticosteroid therapy between second-degree and third-degree AVBs as well as the influencing factor of transient versus recurring occurrences. Indeed, there may be an opportunity to avoid device implantation and its associated procedure-related risks and complications among selected patients.
